# First pilot study for newborn screening of severe T and B lymphopenias in Colombia

**DOI:** 10.7705/biomedica.7568

**Published:** 2024-12-23

**Authors:** Sebastián Gutiérrez-Hincapié, Carlos Muskus-López, Isaura Pilar Sánchez, José Luis Franco-Restrepo, Claudia M. Trujillo-Vargas

**Affiliations:** 1 Grupo de Inmunodeficiencias Primarias, Facultad de Medicina, Universidad de Antioquia, Medellín, Colombia Universidad de Antioquia Universidad de Antioquia Medellín Colombia; 2 Programa de Estudio y Control en Enfermedades Tropicales - PECET, Universidad de Antioquia, Medellín, Colombia Universidad de Antioquia Universidad de Antioquia Medellín Colombia; 3 Grupo de Investigaciones Biomédicas UniRemington, Facultad de Ciencias de la Salud, Corporación Universitaria Remington, Medellín, Colombia Corporación Universitaria Remington Corporación Universitaria Remington Medellín Colombia

**Keywords:** Neonatal screening, lymphopenia, T-lymphocytes, B-lymphocytes, severe combined immunodeficiency., tamización neonatal, linfopenia, linfocitos T, linfocitos B, inmunodeficiencia combinada grave.

## Abstract

**Introduction.:**

Congenital lymphopenias cause increased susceptibility to infections in children apparently healthy at birth. Earlier detection of these conditions would facilitate prompt treatment, prevent potentially serious disease complications and early deaths, and save healthcare resources.

**Objective.:**

To perform a pilot study for neonatal screening of congenital lymphopenias by the quantification of TREC and KREC -T- and B-cell receptor excision circles- in peripheral blood samples from newborns in Medellín, Colombia.

**Materials and methods.:**

Blood samples from 1,092 newborns and six referred patients with suspected lymphopenia were collected by heel or toe-finger prick and dropped onto a filter paper. Thereafter, DNA was extracted and levels of TRECs and KRECs were measured by qPCR.

**Results.:**

The six patients with suspected lymphopenia showed undetectable or very low TREC levels. All newborns screened presented normal TREC and KREC levels. A positive correlation was found between TREC or KREC values quantified from two different filter papers. Detectable levels of the receptor excision circles decrease considerably after 24 weeks of the dried blood spot sample storage. We identified a positive association between low TREC levels and low birth weight; and a negative correlation between KREC values and prematurity. Finally, no statistical differences were found between TREC or KREC levels and delivery method.

**Conclusion.:**

We describe the first preliminary study for the early detection of lymphopenias in Colombia. We proposed to use a cut-off value of 119 and 69 copies/pl blood of TREC and KREC, respectively for future newborn screening programs in our country.

## Resumen

**Introducción.:**

La detección temprana de linfopenias congénitas facilitaría su tratamiento oportuno y la prevención de complicaciones serias, y optimizaría los recursos del sistema de salud.

**Objetivo.:**

Realizar un estudio piloto para el tamizaje neonatal de linfopenias mediante la cuantificación de los círculos de escisión (TREC y KREC) de los receptores de los linfocitos T y B en muestras de sangre periférica de neonatos de Medellín, Colombia.

**Materiales y métodos.:**

Se recolectaron muestras de 1.092 neonatos y de seis pacientes con sospecha de linfopenia, mediante pinchazo del talón o del dedo gordo del pie. Se extrajo el ADN y se midieron los niveles de TREC y KREC por qPCR.

**Resultados.:**

En los seis pacientes con sospecha de linfopenia se encontraron niveles muy bajos o indetectables de TREC. Todos los neonatos tamizados presentaron niveles normales de TREC y KREC. Se observó concordancia entre los valores de TREC o de KREC en dos tipos diferentes de papel de filtro. Los niveles de TREC y KREC disminuyeron considerablemente después de 24 semanas de almacenamiento de las muestras de sangre en papel de filtro. Se encontró una asociación positiva entre los niveles de los TREC y el peso al nacer, y una correlación negativa entre los KREC y la edad gestacional. Los niveles de estos marcadores no se asociaron con el tipo de parto.

**Conclusiones.:**

Se presenta el primer estudio piloto para el tamizaje temprano de linfopenias congénitas en neonatos en Colombia. Se propone el uso de un límite de detección de 119 y 69 copias/pl de sangre de TREC y KREC, respectivamente, para futuros programas de detección neonatal de linfopenias en el país.

Primary immunodeficiency diseases, also known as inborn errors of immunity, are genetic disorders in which components of the immune system are missing or impaired. This alteration could increase susceptibility to infections, autoimmunity, uncontrolled inflammation, and cancer. Recent epidemiological studies have shown that primary immunodeficiency diseases are more common than previously thought and that as many as 1% of the population might be affected ^1^. These estimations increase steadily when programs for their screening and active search are established ^2^.

T cell lymphopenias, characterized by a marked decrease or absence of T lymphocytes in peripheral blood, are considered the most severe primary immunodeficiency disease ^3^^,^^4^. Among them, severe combined immunodeficiency represents a pediatric emergency, with a frequency of 1:58,000 live births. This condition comprises more than 18 monogenic defects associated with impaired maturation and development of T lymphocytes ^5^. However, the immunological phenotype and clinical characteristics vary depending on the presence or absence of B and NK (Natural Killer) cells and the penetrance of the genetic defect.

The diagnosis of inborn lymphopenias is usually delayed because symptoms only start to emerge after the first months of life in children apparently healthy at birth. Thereafter, they develop chronic, debilitating, and often fatal infections. This scenario has a high social and economic impact due to the increasing costs of hospitalization and Intensive care unit-targeted interventions ^6^^,^^7^. One of the most important strategies for the timely care of these conditions is their early detection since immunological restoration through currently available therapies could be promptly undertaken.

In 2005, Chan and Puck ^9^ introduced a severe combined immunodeficiency newborn screening based on the methodology suggested by Douek *et al*. ^8^. This strategy quantifies the DNA byproducts generated after somatic recombination of the T-cell receptor genes, called T-cell Receptor Excision Circles (TREC) ^9^. TREC are episomal DNA circles generated during T-lymphocyte differentiation in the thymus, comprising a progressively random rearrangement of different DNA segments from the variable T-cell receptor chains to form a unique and functional broad- spectrum repertoire. Since TREC do not replicate during T cell division, impairment in the proper maturation and production of T lymphocytes in the thymus would directly correlate with a marked decrease in their production.

After the first severe combined immunodeficiency newborn screening study of TREC in blood spots dried onto filter paper cards was published ^10^, this condition was included in the newborn screening panel in the United States in 2010. Currently, this test is fully implemented in the newborn screening programs of 48 out of 50 states in the USA besides Puerto Rico, with a coverage of 95% of all newborns ^11^. In 2007, van Zelm *et al*. also investigated the replication history of B lymphocytes, quantifying the so-called kappa-deleting recombination excision circles (KREC) ^12^. Studies in Brazil and Germany have reported that TREC and KREC detection in blood spots dried onto filter paper cards has a sensitivity of 100% for the diagnosis of severe combined immunodeficiency, being also time- and cost- effective ^13^^,^^14^. In Colombia, only congenital hypothyroidism is included in a nationwide newborn screening program ^15^. However, a relatively recent law-N° 1980-, approved in 2019, officially recommended expanding the diseases Included in the newborn screening panel (https://www.minsalud.gov.co/Normatividad_Nuevo/Ley%201980%20de%202019.pdf).

The main goal of this study was to perform the first pilot study for the newborn screening of congenital lymphopenias in Colombia, using TREC and KREC quantification in dried blood spot samples collected in filter paper cards.

## Materials and methods

### Screened population

We included newborns from the *Clínica Universitaria Pontificia Bolivariana, Hospital General de Medellín, and Hospital Universitario San Vicente Fundación* (all located in Medellín) and six (P1-P6) referred patients with suspected T or B lymphopenia.

The protocol was approved by the corresponding institutional review boards, following the guidelines of the Declaration of Helsinki. At least one of the parents consented to participate in the study before blood sample collection. Newborn variables such as gender, gestational age, birth weight, and delivery method were collected during the interview with the parents. The number and reason for refusals were also registered for a subgroup of newborns.

Blood samples were collected onto Whatman 903 filter paper cards or Whatman™ filter paper grade 3 by heel or toe finger prick with pediatric lancets (Accu-Chek Safe-T Pro Plus™, Roche, Basel, Switzerland). Specimens were allowed to dry on a flat bench at room temperature for at least three hours and were stored individually at -20 ^2^C in a gas-impermeable zip-lock bag under desiccant conditions until processing. For the lymphopenic patients, the data from lymphocyte subpopulations in peripheral blood were obtained from the clinical records.

### TREC/KREC and β-actin plasmids

The pCR2.1-TOPO vector (Invitrogen, Carlsbad, California, USA) carrying the specific TREC/KREC or β-actin sequence -described by Sottini *et al*. ^16^-was generously donated by the *Instituto de Pesquisa Pelé Pequeno Príncipe* in Curitiba, Brazil. Plasmids were subsequently transformed into DH5-alpha competent cells (New England Biolabs, Ipswich, MA) following the manufacturer’s instructions. Plasmid DNAwas extracted using the PureLink Quick Plasmid Miniprep Kit™ (Invitrogen).

The extracted DNA constructs were sequenced by the Sanger method using the universal primers M13F-GTAAAACGACGGCCAGT and M13R- GCGGATAACAATTTCACACAGG to verify the presence of the specific TREC/KREC or |3-actin inserts. Finally, plasmids were linearized by enzymatic digestion with Xhol restriction enzyme (Thermo Fisher Scientific, Waltham, MA). The digested product was purified using the QIAquick PCR Purification Kit™ (Qiagen, Hilden, Germany) and then used to construct the qPCR standard curve.

### DNA extraction and quantitative real time PCR (qPCR)

Disks of 3.2 mm diameter were punched from the dried blood spot cards and added onto a 96-well polypropylene DNA-RNA-free plate (Applied Biosystems, Thermo Fisher Scientific) to perform the DNA purification and elution procedure, as previously described ^8^^,^^17^.

The extracted DNA was diluted on 24 pi of elution solution (Gentra Generation DNA Elution, Qiagen), and 8 pi were used for the qPCR. A standard curve was constructed using serial dilutions containing 1 x 10^1^ to 1 x 10^6^ copies of the TREC/KREC plasmids and 5 x 10^1^ to 50 x 10^6^ copies of the (3-actin plasmid. All calibration curves had a R^2^ of 0.99.

The qPCR reaction (20 µl) contained 10 pi of TaqMan Gene Expression Master Mix, 0.2 µM of TREC and KREC specific primers, 0.2 µM of the FAM and VIC specific probes ^16^ (both paired with the non-fluorescent minor groove binder quencher NFQ-MGB) respectively, and 8 pi of PCR grade water.

The amplification of (β-actin was performed only for samples with low TREC or KREC values. These values were normalized to microliters of whole blood based on the estimation that each 3.2 mm paper disk contains 3 pi of total blood. Values above 8,000 copies/µl for β-actin quantification were considered an indicator of good quality of DNA extraction ^13^.

The qPCR was performed using a StepOnePlus Real-Time PCR™ system (Thermo Fisher Scientific) with an initial cycle at 50°C for 2 minutes, followed by a cycle at 95°C for 10 minutes and 45 cycles at 95°C for 30 s and 62°C for 30 s. All reagents for the qPCR were purchased from Thermo Fisher Scientific™ (Carlsbad, CA).

### Statistical analysis

Statistical analyses were performed using SPSS™ software (IBM SPSS Statistics for Windows, version 24.0) (Armonk, NY). We applied the Kolmogórov-Smirnov test to evaluate normality. Results are presented as the median and interquartile range (IQR). Statistical comparison between two groups was performed using the Mann-Whitney U test, with a confidence level of 95%. Statistical comparisons among three or more groups were performed using the Kruskal-Wallis test and Dunnett’s T3 post hoc test, with a confidence level of 95%. Spearman correlation coefficient was used for correlation analyses. We considered statistically significant a p value lower than 0.05.

## Results

### Demographic features of the population

Newborns (n = 1,092, 49% male) were recruited from the *Clínica Universitaria Pontificia Boiivariana* (n = 501,46%), *Hospital General de Medellín* (n = 339, 31%) and *Hospital Universitario San Vicente Fundación* (n = 252, 23%). Most newborns included in the study were full-term (> 37 weeks gestational age, n = 693/1,090, 64%) and had normal weight at birth (n = 597/961,62%, > 2,500 g), with a median of 37.6 weeks (IQR = 35 - 39) and 2,790 g (IQR = 2,110-3,215).

Data from delivery methods were collected from 860/1,092 children included, and among them, 69% were born by vaginal delivery (n = 593). The median age of the mothers recruited was 24 years (IQR = 20-30). From a subgroup of 492 mothers interviewed, 10.4% refused to enroll their infants in the study. The reasons for declining participation are presented in table 1.

**Table 1.** Reasons for declining participation in a newborn screening for severe combined immunodeficiency and T or B lymphopenia (51 refused from 492 mothers interviewed)

Reason	n	%
The newborn has received many punctures.	26	51
No specific reasons	10	20
The mother needs to ask the father about it.	4	8
The father refused to participate.	3	6
It is not necessary.	3	6
The baby looks healthy.	2	4
The mothers need to ask the pediatrician.	1	2
The mother is depressed.	1	2
The baby has pneumonia and “it is better not to bother him”.	1	2
Refusals, total	51	100

### Quantification of the TRECs and KRECs in newborns

All samples from the newborns enrolled in the three hospitals showed levels of TRECs and KRECs above 119 and 69 copies/µl, respectively. (table 2). TRECs were consistently higher than KRECs and we did not find a significant correlation between these two measurements. No statistical differences were found between the levels of TREC and KREC in newborns from the three hospitals enrolled.

**Table 2.** Quantification of T-cell receptor excision circles (TRECs) and kappa-deleting recombination circles (KRECs) in newborn samples

	Median (IQR)	Min - Max	95% CI
TRECs (copies/µl)	4,769 (1,884 - 9,592)	119 - 49,082	4,402 - 5,040
KRECs (copies/µl)	983 (540 - 1,926)	69 - 16,040	9,22 - 1,061

### Quantification of TREC and KREC in patients with T-cell lymphopenia

Since we did not detect any patient with lymphopenia within the newborn screening in the recruiting hospitals, we included in the analysis dried blood spot samples from six patients with confirmed lymphopenia, determined by quantification of T, B, and NK cells subpopulations, as positive controls for test validation (table 3).

**Table 3:** Immunophenotyping of lymphocytes subsets and TRECs/KRECs levels from six patients with suspected lymphopenia

	P1	P2	P3	P4	P5	P6
Age	7 months	17 months	5 months	7 months	4 months	29 days
Lymphocytes, total	410(1,800 - 18,700)	1,818(1,400 - 12,100)	651 (1,800 - 18,700)	120(1,800 - 18,700)	286 (3,400 - 12,200)	-
CD3+ (cells/pl)*	2(1,400 - 11,500)	116(700 -8,800)	38(1,400 - 11,500)	23 (1,400 - 11,500)	24 (2,200 - 9,200)	65 (2,200 - 9,200)
CD3+/CD4+ (cells/pil)*	1 (1,000 -7,200)	97 (400 - 7,200)	35(1,000 -7,200)	19(1,000 -7,200)	17(1,600 - 6,500)	38 (1,600 - 6,500)
CD3+/CD8+ (cells/pl)*	0 (200 - 5,400)	20 (200 - 2,800)	1 (200 - 5,400)	4 (200 - 5,400)	6 (300 - 3,400)	25 (300 - 3,400)
CD3+/CD4+/CD8+ (cells/pl)*	0(6,3-190)	1 (2,3 - 88)	0(6,3-190)	1 (6,3-190)	< 1 (14-240)	ND
CD56+/CD16+/CD3-(cells/pl)*	43 (68 - 3,900)	104 (55-4,000)	28 (68 - 3,900)	14 (68-3,900)	144 (97 - 1,990)	13 (97- 1,990)
CD19+ (cells/pl)*	297(130 - 6,300)	1,584 (160 -3,700)	605(130 - 6,300)	29 (130 -6,300)	12(520-2,300)	4 (520 - 2,300)
TREC (cells/pl)	0	0	0	0	0	9
KREC (cells/pl)	254	1,419	1,665	9	28	17
(3-actin (copies/pl)	22,670	8,786	10,419	9,084	9,385	12,162
Phenotype	T-/B+/NK-	T-/B+/NK+	T-/B+/NK-	T-/B-/NK-	T-/B-/NK+	T-/B-/NK-

TREC: T-cell receptor excision circles; KREC: Kappa-deleting recombination excision circles; ND: not available

* Lymphocyte subpopulations: numbers represent absolute counts. Reference values according to age are shown in parenthesis

We had patients with different types of T cell lymphopenias, namely, two patients with T-/B+/NK-, one with T-/B+/NK+, two with T-/B-/NK- and one with T-/B-/NK+ lymphopenias. These results correlated with the absence of TREC levels in the six patients and lower KREC levels (< 30 copies/pl of blood) observed in P4, P5, and P6. In all patients dried blood spot samples, we quantified (β-actin as an indicator of appropriate DNA extraction, obtaining normal values (above 8,000 copies/pl of blood). To evaluate the method of reproducibility, we repeatedly tested dried blood spots from P3 in different runs with no detectable TREC and normal KREC levels.

### Quantification of TREC and KREC in two filter papers and the influence of storage time

**Figure 1.**
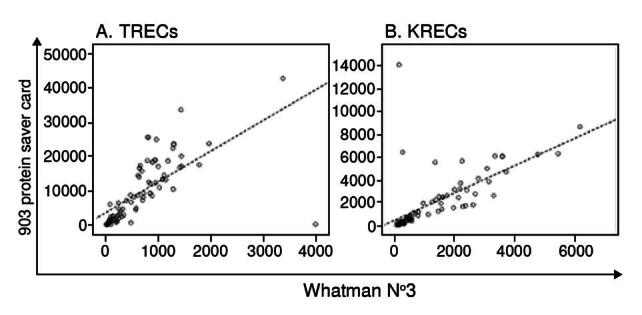
Correlation of TREC and KREC quantification in two different filter papers. Blood spots were obtained from newborn by heel prick and sample delivery in two different filter papers: 903 protein saver card and Whatman N°3. TREC (n = 86) and KREC (n = 79) were quantified. Scatter plots show both measurements.

Considering that differences in the price would make a considerable saving for a nationwide screening of lymphopenias in our country, we consecutively collected dried blood spots from 86 newborns in two different filter papers for TREC quantification. Later, KREC measurements from 79 samples were included in this analysis. Then, we performed a correlation between the excision circles’ quantification in both filter papers. As evaluated with the Spearman coefficient, a positive significant correlation was found for the excision circles quantification in both filter papers (p = 0.846 for TREC and p = 0.854 for KREC; p < 0.05) (figure 1). During sample collection, the only difference we observed between the two filter papers was a faster spread of the blood drops in the Whatman 903™ filter paper cards with a delay time of only a couple of seconds for the Whatman filter paper grade 3.

**Figure 2.**
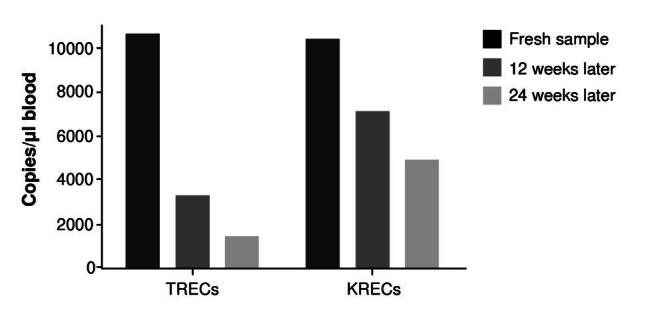
Effect of the sample storage time in TREC and KREC quantification. Bars indicate the number of copies of TREC and KREC per pi in one fresh dried blood spot and the same sample stored for 12 and 24 weeks.

We tested the Influence of short-time storage by analyzing fresh and stored dried blood spots from the same individuals. As shown In figure 2, dried blood spots demonstrated lower TREC or KREC levels, as storage time increased.

### Association of birth weight, gestational age, and delivery method with the blood excision circles values

**Figure 3.**
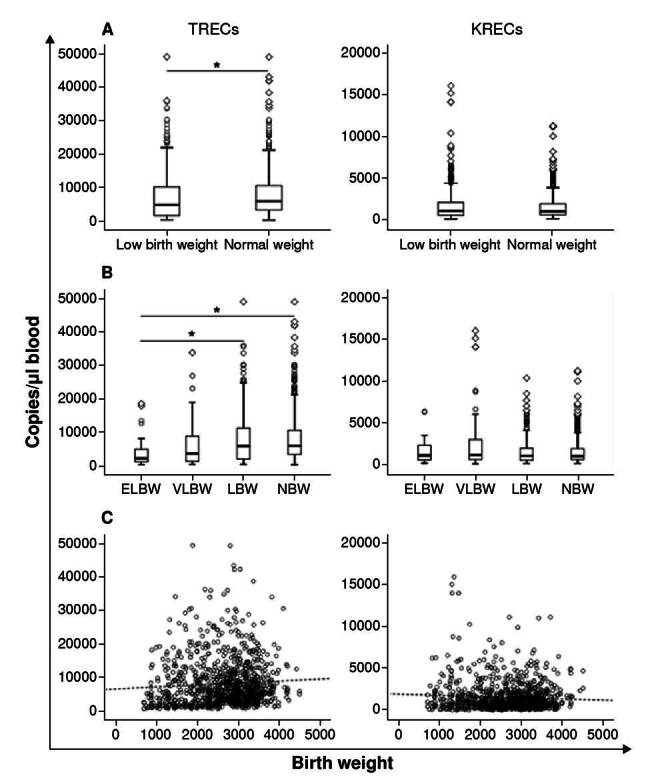
TREC and KREC values according to newborn's birth weight. Box and whiskers plots displaying TREC or KREC levels in dried blood spots from newborns with: A) low (< 2,500 g; n = 364) and normal birth weight 2,500 g; n = 597); B) extremely low birth weight (< 1,000 g, n=29), very low birth weight (> 1,000 to < 1,500 g, n = 86), low birth weight ( > 1,500 to < 2,500 g; n = 249), and normal birth weight (> 2,500 g; n = 597). The Mann-Whitney U test was performed in figure A and the Kruskal-Wallis test in figure B. C) Spearman correlation between TREC or KREC values and birth weight. The dashed line represents the trend. ELBW: extremelylow birth weight; VLBW: very low birth weight; LBW: low birth weight; NBW: normal birth weight * p < 0.05

A total of 961 samples with documented birth weight were analyzed according to TREC or KREC levels. Significantly lower TREC values (Mann- Whitney U test, p < 0.05; figure 3A) were found in newborns with birth weight classified as low (< 2,500 g; TREC median = 4,775 copies/pl; IQR = 1,505 -10,101) compared to those with normal birth weight (TREC median = 5,774 copies/pl; IQR = 3,208 - 10,457).

We also performed a further analysis classifying birth weight in sub-groups according to the World Health Organization (WHO) definition and categories: 29 (3.0%) were born with extremely low birth weight (< 1,000 g), 86 (8.9%) with very low birth weight (> 1,000 to < 1,500 g), 249 (25.9%) with low birth weight (> 1,500 to < 2,500 g), and 597 (62.1%) with normal birth weight (> 2,500 g). Again, TREC values were significantly lower (Kruskal-Wallis test, p < 0.05) in the extremely low birth weight group (median = 2,084 copies/pl, IQR = 972 - 4,746) compared to low birth weight (median = 5,730 copies/pl; IQR = 1,836 - 11,086) and normal birth weight (median = 5,774 copies/pl; IQR = 3,208 - 10,457 copies/pl). KREC values did not vary according to birth weight (figure 3B). However, no correlation was found between the overall birth weight and the levels of excision circles in blood (Spearman p = 0.131 and p = 0.005 respectively (figure 3C).

**Figure 4.**
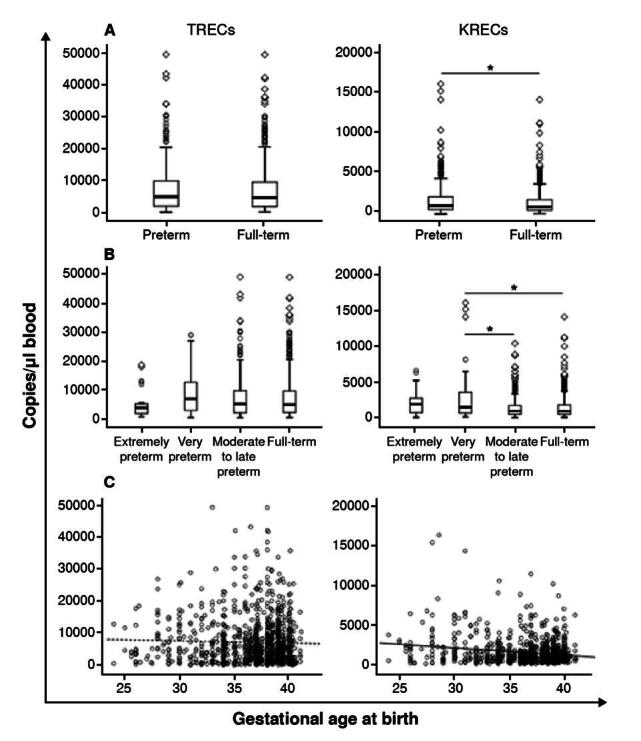
TREC and KREC expression according to newborn's gestational age. Box and whiskers plots displaying TREC or KREC levels in dried blood spots from: A) preterm (< 37 weeks; n = 397) and full-term newborns (> 37 weeks; n = 693); and B) extremely preterm (< 28 weeks; n = 27), very preterm (28 - 31 weeks; n = 82), moderate to late preterm (32 - 36 weeks; n = 288), and full-term newborns (> 36 weeks; n = 693). A Mann-Whitney U test was performed in figure A and a Kruskal-Wallis test in figure B. C) Spearman correlation between TREC or KREC values and gestational age. The dashed line represents the trend.

We analyzed TREC and KREC blood values concerning gestational age. We classified the gestational age Into two groups: Preterm (< 37 weeks) and full-term (> 37 weeks). Although no statistical difference was observed related to the TREC levels (Mann-Whitney U test, p > 0.05; median = 1,078 copies/ pi; IQR = 600 - 2,166), KREC values were significantly higher in premature infants (Mann-Whitney U test, p < 0.05; median = 936 copies/pl; IQR = 530 - 1,820) (figure 4A).

We similarly performed a further analysis using a subgroup classification for gestational age according to the WHO definition: 27 (2.5%) were extremely preterm (< 28 weeks), 82 (7,5%) were very preterm (28 - 31 weeks), 288 (26,4%) were moderate to late preterm (32 - 36 weeks), and 693 (63,6%) were full-term (> 36 weeks). TREC values did not vary according to gestational age. However, KREC levels were significantly higher (Kruskal- Wallis test, p < 0.05) in very premature newborns (median = 1,506 copies/pl; IQR = 707 - 3,563) compared to moderate to late premature (median = 936 copies/pl; IQR = 520 - 1,820 copies/pl) and full-term newborns (median = 970 copies/pl; IQR = 528 - 1,776). No correlation was found between the overall gestational age at birth and the TREC (p = -0.024) or KREC (p = 0.085) blood values (figure 4C).

**Figure 5.**
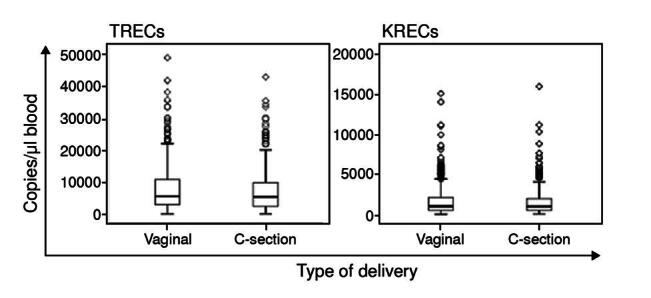
TREC and KREC values according to the delivery method. Box and whiskers displaying TREC (n = 593) or KREC (n = 267) levels in newborns delivered by vaginal or C-section. The Mann-Whitney U test did not show any statistical significance.

Further analyses between TREC or KREC values and delivery method were performed, but no statistical differences were found (Mann-Whitney U test: p > 0.05) (figure 5).

## Discussion

In the present study, we prospectively collected 1,092 dried blood spots from newborns and six from infants with suspected lymphopenia, to quantify blood levels of TREC and KREC. Of the parents interviewed, 10% disagreed with participating in the present study. In a study in the Navajo Nation, 34% of the mothers (959 out of 2,796 mothers interviewed) refused to enroll their infants in the severe combined immunodeficiency newborn screening program ^18^. Parental acceptance of these programs is widely discussed ^19^. Effective parent outreach and communication need to be implemented to understand the reasons for newborn screening testing ^20^.

The TREC assay has been constantly modified worldwide, and several laboratories have adapted their own protocols. A systemic review published by van der Spek *et al*. evaluated scientific literature about protocols used for TREC-based newborn screening worldwide ^21^. Changes among laboratories are generally performed to the DNA extraction method (punch size, elution solution, incubation times, and volumes) and to the quantitative PCR (platform, standards, controls, reagents, primers, and probes).

In our study, we used the protocol suggested by Douek *et al*. and adapted by Baker *et al*. by introducing some changes related to the qPCR ^8^^,^^17^. For example, we used multiplex TaqMan qPCR probes as previous studies did to quantify excision circles ^22^^,^^23^. Regarding the cut-off values, the lower TREC and KREC values found in our study were 119 and 69 copies/pl of blood, respectively.

Our data was validated by analyzing six patients with confirmed T or B lymphopenia according to peripheral blood lymphocyte subpopulation counts. Three of them (P1-P3) presented with the T-/B+/NK variable immunological phenotype and showed undetectable TREC but normal KREC values; the other three patients (P4-P6) had the immunological phenotype T-/B-/NK- and did not show detectable TREC levels but had low KREC values. In all six patients, we assessed β-actin levels, obtaining more than 8,000 copies/µl of blood, as recommended by Kanegae *et al*. ^13^. According to our results, we proposed to use these values as cut-offs for future newborn screening programs in our country. Different studies have established their own cut-offs, and they differ from each other worldwide and even inside the same country ^21^.

We also investigated the association among birth weight, gestational age, and delivery method with TREC or KREC copy numbers in the newborns with available information. Infants with low birth weight presented lower TREC values at birth than those with normal birth weight. About KREC, the values were statistically different between preterm and full-term infants.

Our findings disagree with some reports documenting a positive correlation between gestational age and birth weight with TREC or KREC levels. However, they concord with several reports mentioning reduced levels of TREC and KREC in low-birth-weight newborns ^24^^-^^26^. There is also evidence that prematurity has been associated with abnormal TREC or KREC levels at birth ^13^^,^^27^^-^^29^, even though, in our study, no differences were observed regarding TREC levels and prematurity.

All these differences between studies could be attributed to the high variability of TREC and KREC levels even in newborns of similar gestational age or weight at birth, as reported by Rechavi *et al*. ^25^. Previous studies showed that preterm infants exhibited significantly smaller numbers of T and B lymphocytes probably because of a smaller thymus ^30^^,^^31^.

We also found no association between the delivery method and TREC or KREC values. However, Schlinzig *et al*. reported an association between low TREC values and cesarean section ^32^. In their study, infants born by cesarean delivery showed a 32% higher risk of having a lower number of newly formed T lymphocytes than those born by vaginal delivery. This finding was explained by the absence of contact between the maternal vaginal and intestinal microbiota during cesarean delivery, which plays an important role in the postnatal development of the immune system ^33^^,^^34^.

This study is very relevant due to the tremendous importance of severe combined immunodeficiency newborn screening for public health, especially in low- and middle-income countries ^35^^,^^36^. Infants with abnormal TREC- newborn screening are at a higher risk of morbidity and mortality, especially if they are sent home before a comprehensive study of the causes of immunodeficiency ^37^. A recent study from Australia found that universal newborn screening for severe combined Immunodeficiency would gain ten quality-adjusted life-years at a cost of USD $0.3 million ^38^. Also, improved severe combined immunodeficiency screening recommendations for premature and low birth weight populations have been reported ^29^.

Our study demonstrates that TREC, KREC, and (3-actin quantification in newborn screening is an effective strategy to detect T or B lymphopenias. We proposed to use the minimum values detected in all assessed patients (119 TREC copies/pl and 69 KREC copies/pl) for future newborn screening programs in Colombia. Greater efforts are still required to persuade clinicians and local healthcare providers about the importance of carrying out newborn screening programs in our country to facilitate the early treatment of patients with these threatening conditions.
